# Supplementary material for: Eco-Friendly Silver Nanoparticles Synthesis Method Using Medicinal Plant Fungal Endophytes—Biological Activities and Molecular Docking Analyses
Source: Biology (Basel). 2025 Jul 28;14(8):950. doi: 10.3390/biology14080950 (PMC12383769; doi:10.3390/biology14080950)
Supplement: Supplementary file 1 [file biology-14-00950-s001.zip › biology-3542937-supplementary.pdf]

Supplementary Materials

Tables

Table S1. Computed Physiochemical Parameters of the Dimethylsulfoxoniumformylmethylide.

Physicochemical properties of DMSF	
Formula	$C_4H_8O_2S$
Molecular weight	120.17g/mol
Num. heavy atoms	7
Num. arom. heavy atoms	0
Fraction Csp3	0.50
Num. rotatable bonds	1
Num. H- bond acceptors	2
Num. H- bond donors	0
Molar Refractivity	32.13
TPSA	42.52A^2
**Lipophilicity**	
Log $P_{o/w}$ (iLOGP)	0.00
Log $P_{o/w}$ (XLOGP3)	-0.70
Log $P_{o/w}$ (WLOGP)	0.40
Log $P_{o/w}$ (MLOGP)	-0.53
Log $P_{o/w}$ (SILICOS-IT)	0.29
Consensus Log $P_{o/w}$	-011
**Water Solubility**	
Log S (ESOL)	-0.08
Solubility	1.00e+02 mg/ml; 8.35e-01 mol/l
Class	Very soluble
Log S (Ali)	0.28
Solubility	2.30e+02 mg/ml;1.92e+00 mol/l
Class	Highly soluble
Log S (SILICOS-IT)	-0.62
Solubility	2.88e+01 mg/ml; 2.40e-01 mol/l
Class	soluble
**Pharmacokinetics**	
GI absorption	High
BBB permeant	No
P-gp substrate	No
CYP1A2 inhibitor	No
CYP2C19 inhibitor	No
CYP2C9 inhibitor	No

CYP2D6 inhibitor	No
CYP3A4 inhibitor	No
Log K_p (skin permeation)	-7.53cm/s
**Druglikeness**	
Lipinski	Yes; 0 violation
Ghose	No; 3 violations: MW<160, MR<40,# atom<20
Veber	Yes
Egan	Yes
Muegge	No; 2 violations: MW<200,# C<5
Bioavailability Score	0.55
**Medicinal Chemistry**	
PAINS	1 alert: thio_aldehyd_A
Brenk	2 alert: aldehyde, thiocarbonyl_group
Leadlikeness	No; 1 violation: MW<250
Synthetic accessibility	2.61

Figures S1-S11

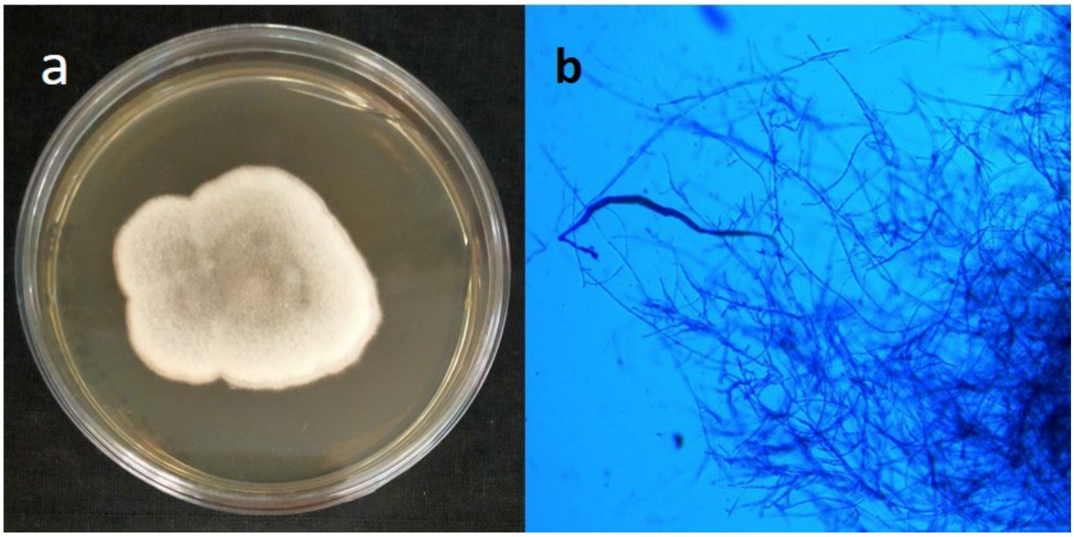

Figure S1. Morphology of *Corynespora smithii* (a) Macroscopic morphology (28 °C, 7days); (b) microscopic morphology (× 400).

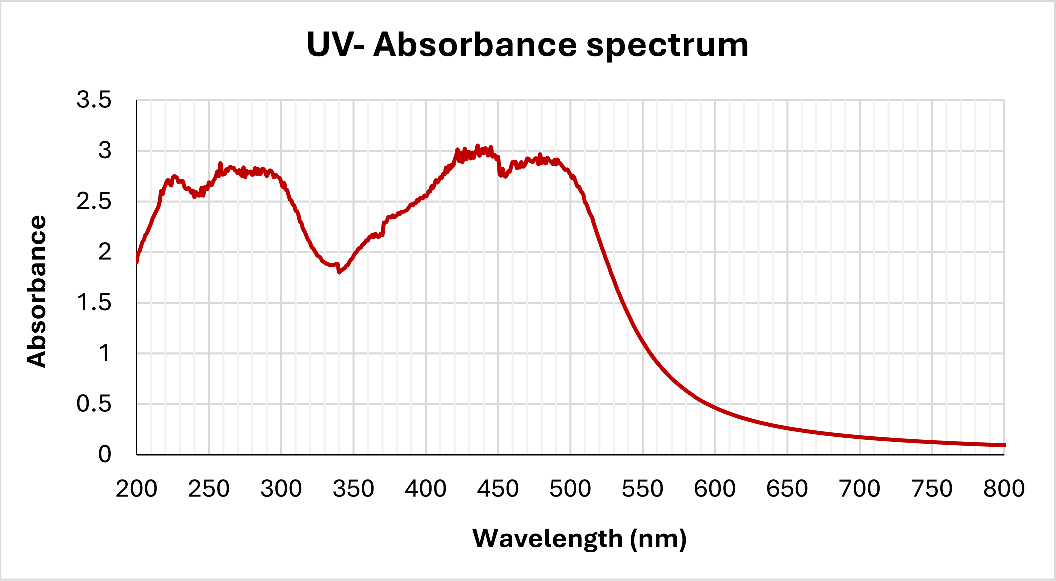

Figure S2. UV Visible spectrophotometry of the fungal mediated synthesized silver NPs.

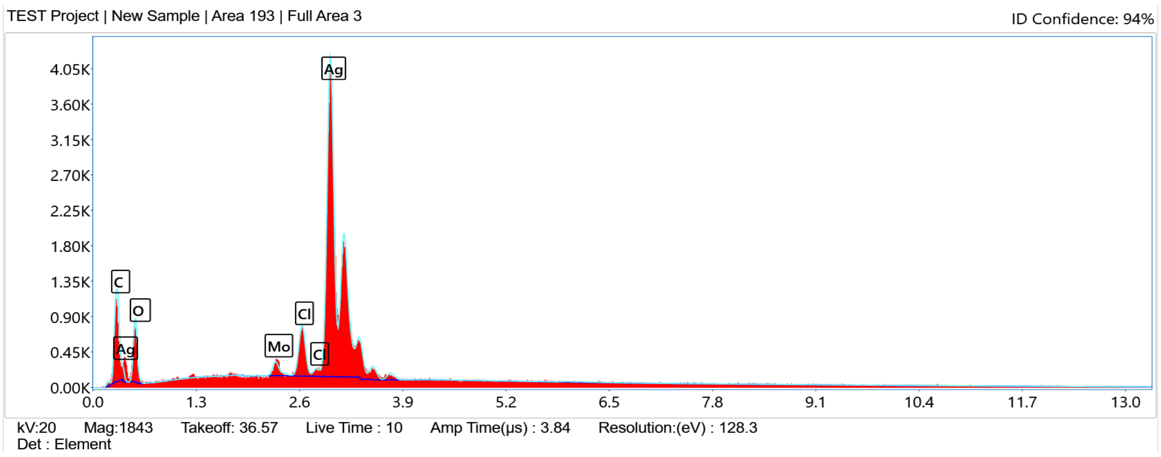

Figure S3. The fungal-assisted silver NPs energy dispersive spectra.

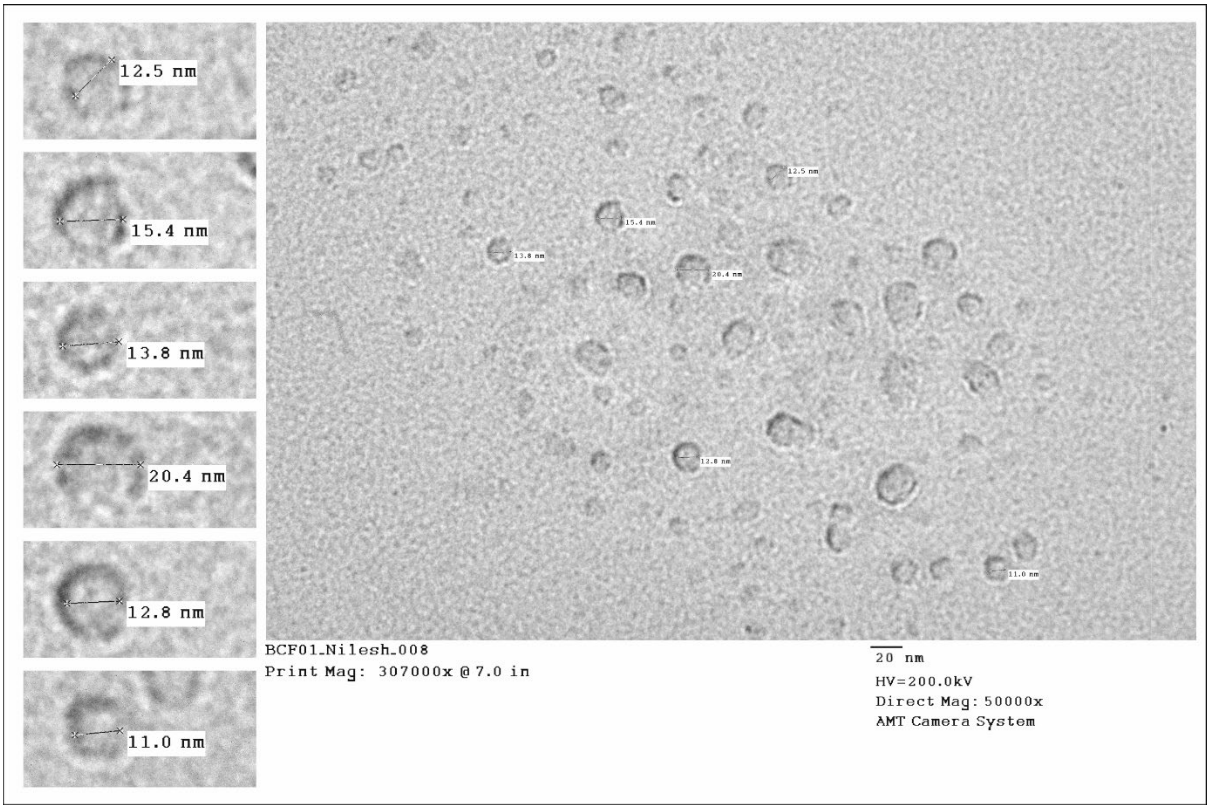

Figure S4. The TEM micrograph of AgNPs synthesized by reduction of silver nitrate with the cell filtrate of *C. smithii*.

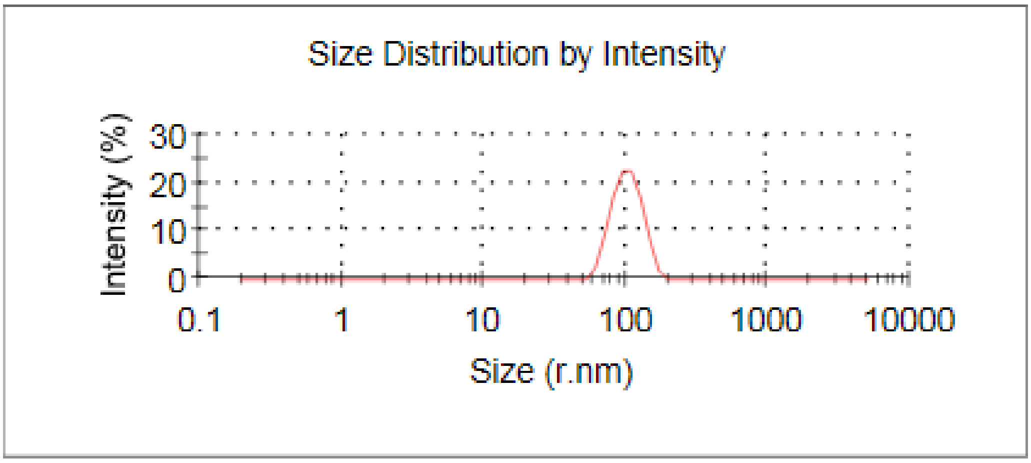

Figure S5. Size distribution of silver nanoparticles synthesized by *C. smithii.*

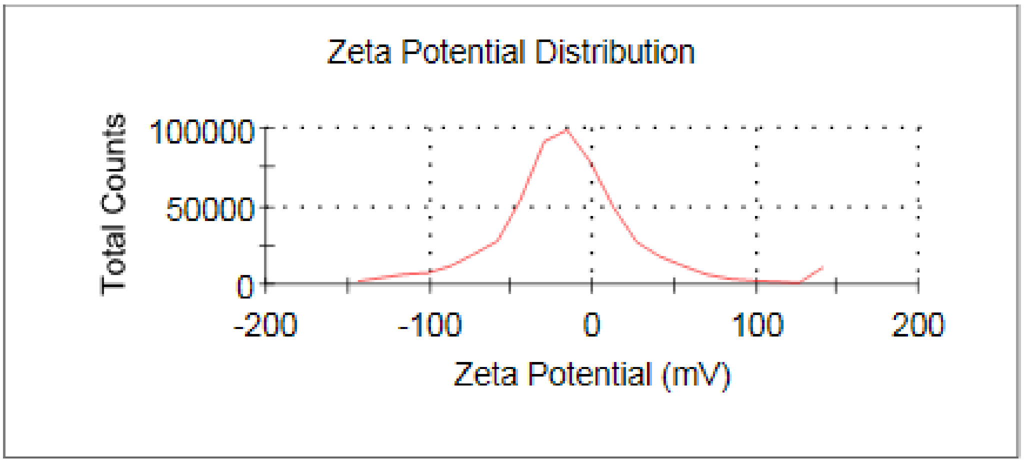

Figure S6. Zeta potential distribution of silver nanoparticles synthesized by *C. smithii.*

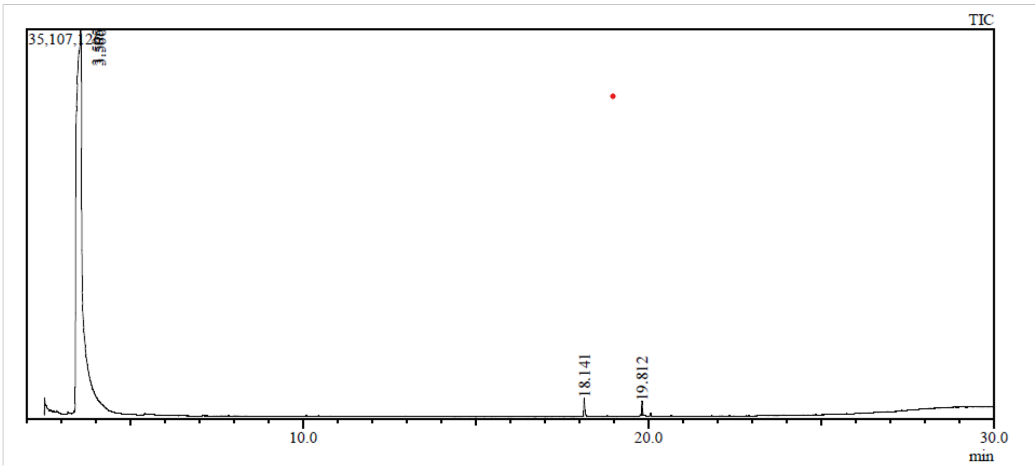

Figure S7. GC MS chromatogram of Fungal assisted Silver Nanoparticle.

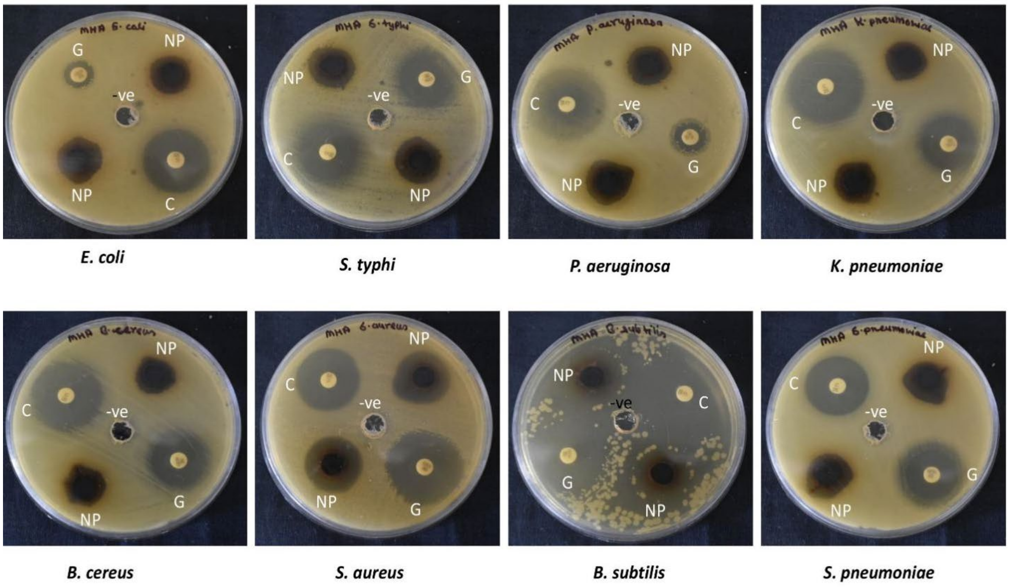

Figure S8. Antibacterial activity of synthesized FANPs against tested bacteria.

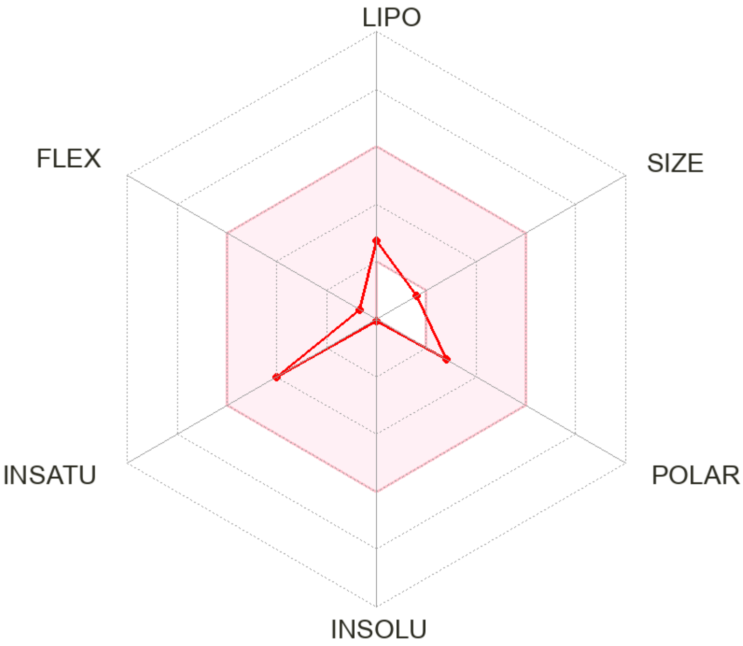

Figure S9. Bioavailability radar plots of DMSF. The colour zone is the suitable physicochemical space for oral bioavailability. LIPO = lipophilicity (−0.7 < XLOGP3 < +5.0), SIZE: 150 < MV < 500 g/mol, POLAR = polarity (20 Å^2< TPSA < 130 Å^2), INSOLU = Insolubility (not higher than -6 < Log S (ESOL) < 0), INSATU = Instauration (0.25 < Fraction Csp3 < 1), FLEX = Flexibility (0 < no of rotatable bonds < 9).

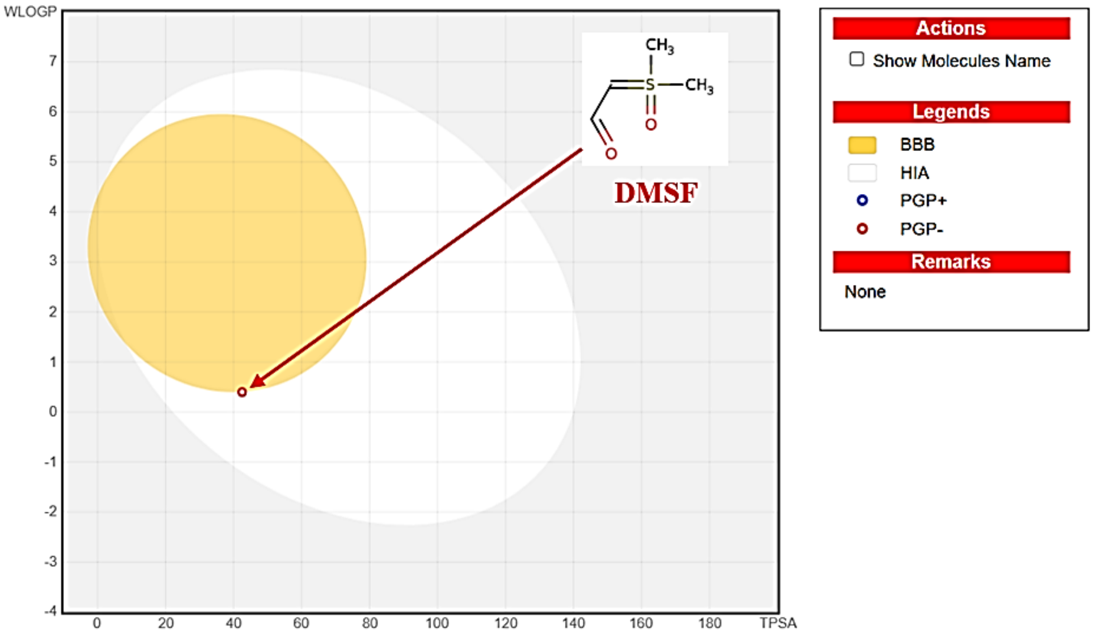

Figure S10. Boiled- egg plot between lipophilicity (WLOGP) and polarity (TPSA) showing DMSF physicochemical properties favorable for both blood-brain barrier (BBB) penetration and high intestinal absorption (HIA).

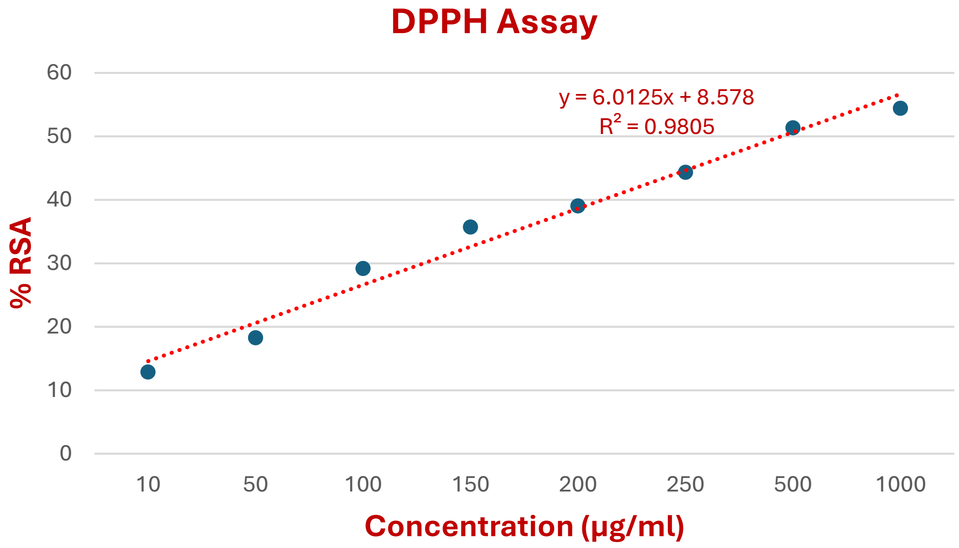

Figure S11. Antioxidant activity of synthesized AgNPs by *C.smithii.*
